# Can Sacrificial Feeding Areas Protect Aquatic Plants from Herbivore Grazing? Using Behavioural Ecology to Inform Wildlife Management

**DOI:** 10.1371/journal.pone.0104034

**Published:** 2014-07-31

**Authors:** Kevin A. Wood, Richard A. Stillman, Francis Daunt, Matthew T. O’Hare

**Affiliations:** 1 Centre for Ecology and Hydrology, Edinburgh, United Kingdom; 2 Faculty of Science & Technology, Bournemouth University, Dorset, United Kingdom; University of Sydney, Australia

## Abstract

Effective wildlife management is needed for conservation, economic and human well-being objectives. However, traditional population control methods are frequently ineffective, unpopular with stakeholders, may affect non-target species, and can be both expensive and impractical to implement. New methods which address these issues and offer effective wildlife management are required. We used an individual-based model to predict the efficacy of a sacrificial feeding area in preventing grazing damage by mute swans (*Cygnus olor*) to adjacent river vegetation of high conservation and economic value. The accuracy of model predictions was assessed by a comparison with observed field data, whilst prediction robustness was evaluated using a sensitivity analysis. We used repeated simulations to evaluate how the efficacy of the sacrificial feeding area was regulated by (i) food quantity, (ii) food quality, and (iii) the functional response of the forager. Our model gave accurate predictions of aquatic plant biomass, carrying capacity, swan mortality, swan foraging effort, and river use. Our model predicted that increased sacrificial feeding area food quantity and quality would prevent the depletion of aquatic plant biomass by swans. When the functional response for vegetation in the sacrificial feeding area was increased, the food quantity and quality in the sacrificial feeding area required to protect adjacent aquatic plants were reduced. Our study demonstrates how the insights of behavioural ecology can be used to inform wildlife management. The principles that underpin our model predictions are likely to be valid across a range of different resource-consumer interactions, emphasising the generality of our approach to the evaluation of strategies for resolving wildlife management problems.

## Introduction

How to manage wildlife effectively for conservation, economic, and human well-being objectives poses a central challenge to humanity [1], [2], [3]. Ineffective management can result in species extinctions and biodiversity loss, reduced ecosystem functioning and service provision, loss of harvestable resources such as food crops, timber and game, outbreaks of agricultural pests and increased human mortality [4], [5], [6]. Thus the consequences of ineffective management can be ecological, economic, aesthetic and social. Traditional attempts to manage animal species that are having such impacts, typically referred to as ‘nuisance’ species, have often focused on controlling the numbers of individuals within defined areas [7]. A range of population control methods have been developed, such as lethal control of individuals [8], scaring and deterrents [9], control of reproduction [10], and translocation of individuals away from the management area [11].

However, several problems with population control methods have been identified. In particular, control can be offset by immigration and increased productivity and survival, and thus population control has been found to be ineffective in a range of systems [7], [12], [13]. Populations may recover rapidly following the cessation of management, and thus population control may not represent a sustainable long-term management plan. Non-target species may also be affected [7]. Concerns regarding animal welfare and the ethics of capturing and killing individuals mean that population control methods can be unpopular with some stakeholder groups [7]. Such opposition can result in legal challenges and non-cooperation from stakeholders which can make it impractical, expensive and time-consuming to implement population control strategies. Even where social and political obstacles do not prevent implementation, the financial and labour costs may prove prohibitive [12]. Therefore a range of wildlife management problems exist which cannot be managed effectively through traditional population control methods.

One seemingly intractable wildlife management problem has been the ecological and economic damage caused by grazing by high abundances of large, herbivorous animals such as ungulates and waterfowl [4], [14], [15]. Such animals can cause damage to natural and agricultural plant assemblages through consumption, trampling and faecal deposition [15]. Whilst such herbivores may also cause increased abundance of natural and agricultural plant assemblages, negative effects are more commonly reported [16]. There is evidence that such grazing conflicts are becoming more intense and widespread due to recent large increases in the population sizes of many waterfowl and ungulate species [14], [17]. Thus there is a need to manage grazing conflicts to reduce ecological and economic damage. However, herbivores may be popular with stakeholders and many have high degrees of legal protection, and so population control can prove difficult to implement. Even where it is possible to implement, population control methods are often ineffective in protecting plant communities, due to high immigration and survival rates [13], [17]. Therefore there is a need for alternative management options that are legal, sensitive to stakeholders and effective in reducing grazing damage within affected areas.

Research into behavioural ecology has provided powerful explanations for observed patterns of animal behaviour and decision making, including the use of foraging habitats [18]. Foragers move between different feeding locations and food resources in order to maximise their perceived fitness. Due to the difficulty of measuring lifetime reproductive success, net rate of energy gain whilst foraging is commonly used as a proxy for fitness; a wide range of studies have demonstrated that differences in net energy gain can explain patterns in resource-consumer interactions, such as animal exploitation of feeding habitat [19], [20]. Insights based on net rates of energy gain have proved particularly robust for highly mobile animals which feed on immobile food resources, such as vertebrate herbivores consuming plant tissues [20]. Animal net rates of energy gain are strongly influenced by the intake rate of foragers, food quantity and food quality, with increases in all three variables resulting in greater rates of gain [18]. Understanding the factors which influence habitat use suggests the possibility of manipulating these factors to modify animal distributions to meet wildlife management objectives [21]. In particular, manipulating the net rates of energy gain within a landscape through habitat modifications could offer an ethical and effective means of resolving conflicts with herbivores, compared with traditional population control.

The provision of alternative food resources, typically within a designated sacrificial feeding area (SFA) created through the modification of existing habitat, has been proposed as a management strategy for a range of wildlife management problems [22], [23], [24], [25], [26]. The food within the SFA is intended to draw individuals of the target species away from the area of conflict. SFAs do not involve killing or capturing wildlife and so are more acceptable to some stakeholder groups than traditional population control methods. As such, SFAs are a promising wildlife management tool for species which are legally protected and popular with the public and special interest groups. SFAs could be particularly effective for large vertebrates which can disperse easily between feeding areas within a landscape, such as herbivores responsible for grazing conflicts [12], [27], [28]. Sowing different plant species and varieties, cutting and grazing, and the application of fertiliser, can each be used to manipulate the quantity and quality of food available within the SFA to the foraging herbivores [29]. The sowing of different plants will also affect herbivore rates of consumption through differences in the functional responses [30]. However, the effects of changes in forager intake rate or food quantity and nutritional quality on species use of SFAs are poorly understood. Consequently we lack a mechanistic, process-based understanding of how such factors influence SFA efficacy, which represents a major barrier to the evaluation of SFAs as a wildlife management tool.

Conducting field trials is arguably the most powerful way to test the effectiveness of new wildlife management strategies, yet such tests can be impractical under certain conditions due to logistical, financial, and ethical issues [31]. In particular, it may be difficult to gain legal approval and stakeholder support for such trials, particularly where the target species is charismatic or the habitat of high value. The use of ecological models offers a means of predicting the effects of management in a fraction of the time, and with none of the practical difficulties associated with field trials [32], [33]. Individual-based models (IBMs) predict the movements and behaviours of animals on the basis of simple behavioural rules, principally that individuals attempt to maximise their perceived fitness [33], [34]. IBMs have provided both a framework with which to test our understanding of animal behavioural decisions, and a means of making predictions of the effects of wildlife management strategies [34], [35], [36]. Field trials may be subsequently conducted for only those wildlife management methods predicted to be most effective.

In this study we assessed whether SFAs, comprised of an area of terrestrial vegetation adjacent to aquatic habitat, could prevent a conservation conflict which currently occurs in some shallow aquatic ecosystems. In such ecosystems, the aquatic plant community is of high conservation value as it fulfils a wide range of roles. Aquatic plants increase and diversify the habitat available for other species including animals and algae, promote stable hydrological regimes and physicochemical conditions, and as both living and decayed tissues offer a key food resource [37]. Consequently, aquatic plant communities are typically designated conservation protection, but are sensitive to a range of perturbations. A number of studies from Europe and North America have demonstrated that grazing by flocks of non-breeding mute swans (*Cygnus olor* Gmelin, 1789), a generalist avian herbivore [38], [39], can damage aquatic plant communities of high conservation value [40], [41], [42]. In particular, mute swan grazing has been reported for shallow river ecosystems of southern England [42], [43], [44]; such grazing conflicts with a key conservation objective for such shallow rivers, the protection of the aquatic plant community which is designated for its high conservation value under the European Union Habitats Directive (92/43/EEC). The biological productivity and conservation status of these lowland river ecosystems is strongly determined by the aquatic plant community, and thus even small reductions in plant abundance can have negative effects on the ecosystem [44]. Reported decreases in aquatic plant biomasses have ranged from 0 to 100% [42], yet even relatively small decreases in biomass reduce the habitat, as well as cover from flow and predators, available for other species [43], [44]. In this region mute swans are non-migratory [45], and feed in the river between May and October, and in adjacent pasture fields between November and April [45]. Management is needed to prevent grazing damage to the aquatic plant community, but catchment-scale population control has been shown to be ineffective and is controversial due to swans popularity and protected status [13], [46]. Furthermore, grazing damage is highly localised in space and time, suggesting that more localised management may be more appropriate. Grazing by flocks of swans affects <0.5 km reaches of river, and only affects a minority of river sites, typically for short periods (<6 weeks) before the flock moves on [44]. Previous research has shown that this pattern of swan habitat selection is determined by changes in the relative profitability of different feeding areas within the landscape [47]. Swan grazing damage to river macrophytes is a particular problem between early-May, when the swan flocks enter the river [47], and mid-June when most individuals move to the estuary to moult [45]. Thus river managers require a solution which prevents localised grazing damage to river plants in early summer, and which is compatible with the status of the mute swan within the UK as a legally protected species popular with the public and many stakeholder groups. Swan habitat selection has been shown to be strongly determined by the relative profitability of river and adjacent pasture habitat, and so SFAs have been identified as a promising management option [45], [47]. Furthermore, conflicts between mute swans and agriculture have been successfully managed with SFAs [48]. A previous study has found that SFAs are a cost-effective option for managing waterfowl grazing conflicts in the UK, compared with population control, compensation schemes or no management [12]. Therefore in this study we used an individual-based model to predict the effectiveness of SFA creation on a conservation objective: the prevention of damage to an aquatic plant community in a UK shallow river catchment by grazing swans. Our hypothesis was that the provision of terrestrial vegetation in an SFA would prevent depletion of aquatic plant biomass in an adjacent section of river. To address this hypothesis, firstly we validated the model predictions against observed field data and assessed the sensitivity of model predictions to changes in parameter values. Then we evaluated how SFA efficacy was affected by (i) food quantity, (ii) food quality, and (iii) forager functional response.

## Methods

### Study system

The River Frome (Dorset, UK) is a mesotrophic chalk river that flows through a catchment dominated by pastoral agriculture. The pasture grass community is dominated by three species; perennial ryegrass (*Lolium perenne* L.), creeping bentgrass (*Agrostis stolonifera* L.) and Yorkshire fog (*Holcus lanatus* L.) [45], which are consumed by swans [49]. The aquatic plant community is dominated by stream water crowfoot (*Ranunculus penicillatus ssp. pseudofluitans* (Syne) S.D. Webster) which is also consumed by swans [42], [43]. Aquatic plants show strong growth between March and May, typically reaching peak biomass by July, before showing a seasonal decline thereafter [42]. Aquatic plants exhibit high spatial heterogeneity in both biomass and two-dimensional cover [42]. Abundance is known to be influenced by a number of biotic and abiotic variables including swan grazing, riparian shading, water temperature and water velocity [42]. The aquatic plant community is protected under the European Union Habitats Directive (92/43/EEC), and the River Frome has been designated a Site of Special Scientific Interest (SSSI) due to its conservation value. We studied a 1.1 km long river reach surrounded by pasture grass fields at East Stoke (50°41′N, 02°11′W). Mute swan grazing of aquatic plants has been reported previously for this site [35], [43], and thus we considered it an appropriate study area in which to address the issue of swan grazing management. Swan grazing damage is highly localised in space and time [42], [43], [44], and this was reflected in our choices of study area size and duration. Grazing by flocks of swans typically affects <0.5 km reaches of river, and only affects a minority of river sites, typically for short periods (<6 weeks) before the flock moves on [44]. A previous study which evaluated swan grazing management for an entire river catchment concluded that such large-scale management was ineffective and recommended testing smaller-scale solutions [13]. Thus river managers require a solution which prevents localised grazing damage to river plants at key river sites. A study area of 1.1 km length of river enabled us to evaluate such a localised management option. We selected the 22 day period between May and June because swan grazing damage to river macrophytes is a particular problem during this period; swan flocks enter the river in May and most individuals move to the estuary to moult in mid-June [45], [47]. Thus both of study area size and duration were appropriate to our study objective.

### Model: overview

We adapted an existing model of a swan population in a river ecosystem [35], which was created using the MORPH IBM [50]. MORPH is a flexible IBM which makes few species- or system-specific assumptions and has thus been used extensively to evaluate the responses of foraging animals to changes in their environment [50]. We adapted the original model to give a more detailed, realistic treatment of swan energetic and foraging parameters (**Table 1**). We parameterised our model for a 1.1 km length of the River Frome and an adjacent pasture field for a 22 day period from 22^nd^ May to 12^th^ June, which represents typical flock usage of a site during the swan grazing period [43]. In MORPH the model world contains a population of individual animals (‘foragers’), who can move between discrete areas (‘patches’) which contain food ‘resources’ which the foragers consume [50]. As the model was parameterised for one social group of a single species and we lacked measures of inter-annual variability for many key parameters, all model simulations were deterministic and thus only a single simulation was required for each set of parameters.

**Table 1:** The values associated with each parameter in the model.

Parameter	Value	Units	Derivation
Initial number of swans	41	Individuals	Peak count reported for study area [43]
Swan metabolic cost of river feeding	392.4	kJ hr^−1^	Cost of river foraging given a water velocity of 0.67 m s^−1^ [47]
Swan metabolic cost of pasture feeding	169.2	kJ hr^−1^	Multiple of BMR given for Bewick’s swan (1.2; [59])
Swan metabolic cost of resting	140.4	kJ hr^−1^	= (*V*O_2_ · *m*) · *e*; where *V*O_2_ was basal oxygen consumption (1.82 · 10^−4^ ml O_2_ g^−1^ s^−1^; [73]), *m* was mean swan mass (10800 g; [38]) and *e* was oxygen energy yield (0.02 kJ ml^−1^ O_2_; [59])
Swan energy store	150920	kJ	The difference between mean body mass and lean body mass (10800–6400 g; [38]), multiplied by the energy content of avian tissue (34.3 kJ g^−1^; [56])
Initial water crowfoot biomass instudy area	185	g DM m^−2^	[43]
Initial water crowfoot biomassoutside study area	171	g DM m^−2^	[43]
Water crowfoot growth rate	0.0	g m^−2^ hr^−1^	Growth rate under swan grazing pressure as swans remove growth tissues [43]
Water crowfoot gross energy content	13.4	kJ g^−1^ DM	[47]
Water crowfoot metabolisability	0.44	Proportion	[47]
Swan functional response foraquatic plants	*I* = ((0.003 · *B*)/(1+ (0.0934 · *B*))) · 3600	g DM hr^−1^	Swan intake rate *I* when feeding on aquatic plant biomass *B* [47]
Initial grass biomass	406	g DM m^−2^	This study
Grass growth rate	0.0	g m^−2^ hr^−1^	This study
Grass gross energy content	15.8	kJ g^−1^ DM	[47]
Grass metabolisability	0.21	Proportion	[47]
Swan functional response forpasture grass	*I* = (((3.6 · (1.38 · 10^−3^ ·(0.0238 · *B*)))/(3.6 ·0.02+ (1.38 · 10^−3^ ·(0.0238 · *B*)))/60) ·1.6) · 3600	g DM hr^−1^	Swan intake rate *I* when feeding on pasture grass biomass *B* [47]

### Model: global parameters

Global parameters were those which set general rules which applied to the entire model, including all patches, resources and foragers. The model ran in hourly time-steps for 22 days [43]. Based on the times of dawn and dusk at our site we distinguished between daylight (06∶00–20∶00), when foraging was permitted, and darkness (21∶00–05∶00), when birds were not permitted to forage as field evidence suggests mute swans do not feed at night [51], [52].

### Model: patch parameters

The model world is comprised of discrete areas called patches. Our model consisted of two patches, a river patch (9153 m^2^) and a pasture field patch (95000 m^2^). The patch sizes were set as 100% of the size of the river channel and field adjacent to the river, respectively, at our study location. Thus, a patch consisted of the total available contiguous area of that habitat type. These patches were adjacent and the birds could move freely between them within a single time step, as has been observed at the site [43]. The birds could also emigrate to the river outside of the model, which was assumed to have equal aquatic plant foraging costs, aquatic plant energy content and metabolisability, but a lower dry matter biomass (171 g DM m^−2^) as reported previously [43].

### Model: resource parameters

**Figure 1:**
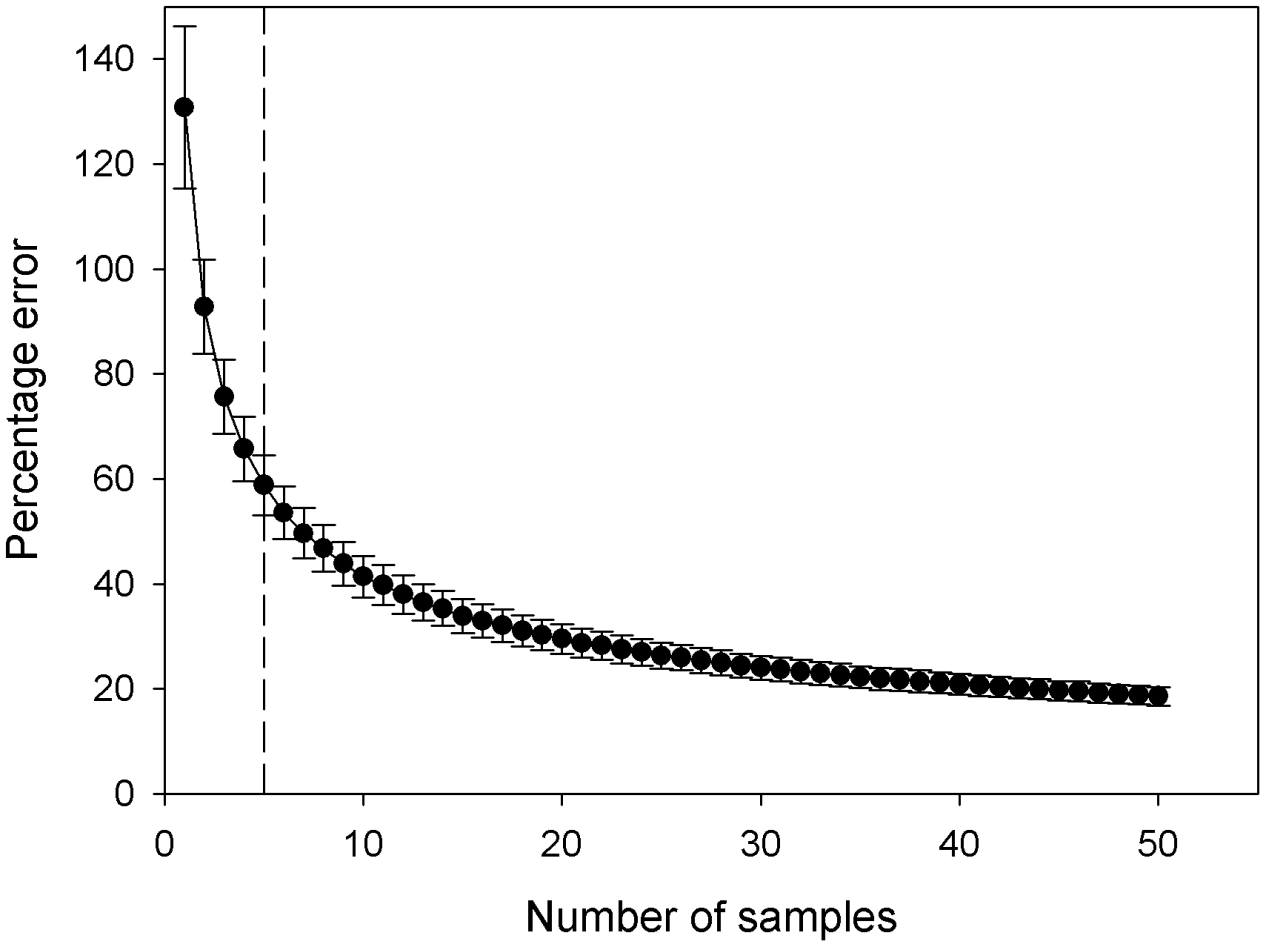
The mean ±95% CI percentage error associated with our estimates of mean pasture grass biomass (g DM m^−2^) for a given number of samples. The dashed line indicates the selected sample size of *n* = 5.

Within each patch are the food resources; in our model there were two resources available to foraging swans, aquatic plants in the river patch and pasture grass in the field patch and SFA. Initial aquatic plant biomass, growth rate over the study period, and the aquatic plant biomass outside of the study area, were those given previously (**Table 1**) [43]. As the river at our site was <1 m deep during our study period [47], [53] and mute swans can reach down to 1 m below the surface [54], we were confident that 100% of aquatic plant biomass was available to swans. To determine the sample size required to estimate pasture grass biomass, in February 2010 we undertook intensive sampling of 20 pasture fields around East Stoke (50°41′N, 02°11′W). Within each field 50 samples were taken, using a 0.00785 m^2^ hand corer commonly used to sample vegetation biomass [42], [53]. We used a randomised sampling strategy to select core sites, whereby vegetation cores were taken from 50 randomised sets of co-ordinates within each field. Bootstrap resampling with replacement was used to derive the relationships between sample size and accuracy of measuring mean pasture grass biomass. For each analysis, *n* samples were selected randomly from the datasets of abundance samples (g DM m^−2^) and the mean was calculated. 10,000 iterations of this process generated a frequency distribution of mean biomass values derived from a sample size of *n*, from which the mean and 95% confidence intervals were calculated, where RCI was the range between the lower 5 and upper 95 percentiles of the Bootstrap frequency distribution. We calculated the percentage error of our biomass measurements by calculating RCI as a percentage of the mean biomass for a given value of *n*; data from all sites were pooled to yield mean (±95% CI) values. Error decreased as sample size increased, but did not decrease below ±18.6% even where *n* = 50 (**Figure 1**). As the greatest decrease in error occurred as *n* increased from 1 to 5 we selected *n* = 5 for quantification of pasture grass biomass, as a compromise between accuracy and sampling effort. Therefore, to estimate pasture grass biomass at our model study site five cores were taken in May and June 2010 from the pasture field at East Stoke, using a 0.00785 m^2^ hand corer and the methodology described above. All above-ground biomass was removed, dried to constant weight at 60°C in a Heraeus Kelvitron T oven (Thermo Fisher Scientific, Loughborough, UK), and weighed to ±0.01 g on a Sartorius PT120 balance (Sartorius GmbH, Germany). Mean dry matter (DM) grass biomass was thus estimated at 406.0 g DM m^−2^ (**Table 1**), and grass biomass change over time (in the absence of swan grazing) was set to 0.0 g m^−2^ hr^−1^, as a T-test indicated no significant difference between grass biomass in May (mean 396.7 g DM m^−2^±251.6 s.d.) and June (mean 415.3 DM g m^−2^±219.1 s.d) (*T* = −0.24, d.f. = 34, *p* = 0.814). The lack of detectable change in grass biomass was probably due to the presence of cattle (*Bos primigenius* L.) in the field over the study period; intensive cattle grazing is known to prevent increases in grass biomass within temperate lowland pasture fields [55]. Gross energy content for pasture grass and water crowfoot were those given previously [47] for the River Frome in May, whilst proportional metabolisability values for swans feeding on pasture grass and aquatic plants were those given previously [47].

### Model: forager parameters

We modelled a flock of 41 non-breeding swans with all individuals present from the first time step and no immigration, based on the population size and dynamics reported previously for our study system [43]. At the beginning of each time step each swan could choose to rest or forage on either aquatic plants or pasture grass for the duration of that time step. Swans consumed their food resources according to the reported functional responses for aquatic plants and pasture grass [47]. Swans in the model were assumed to maximise their net rate of energy gain whilst foraging to maintain their internal energy store at a value of 150920 kJ; estimated as the energy content of avian tissue (34.3 kJ g^−1^; [56]) multiplied by the difference between the mean mass and mass at starvation (10800–6400 g; [38]). Once swans had achieved an energy store above 150920 kJ, and during the hours of darkness, they switched from an energy-maximising to a time-minimising strategy [57]. Swans were assumed to have starved if this energy store was depleted to 0; a starvation event was recorded by the model and the forager concerned was removed from the model. If a swan could obtain a higher net energy gain in the river area outside of the model it would emigrate permanently. Individuals that has emigrated could not re-enter the river area of the model. Thus swans could consider the profitability of the model patches against the profitability of the wider environment. All individuals were designated as non-breeding adults based on the information presented previously [43].

### Testing the model against field data

We tested the accuracy of our model in predicting five properties of the swan grazing system for which field data existed; (i) the carrying capacity of the study area (i.e. both patches combined) expressed as the number of swans multiplied by the number of days each swan was present within the study area, referred to as swan days [43]; (ii) the water crowfoot biomass in the river patch at the end of the simulation, which was a measure of depletion by swan grazing [43]; (iii) the percentage of swan days within both patches that were spent in the river patch, which was a measure of the relative use of river habitat [43]; (iv) the survival probability of swans [43]; (v) the percentage of total time each day which swans spend feeding [58].

### Model robustness

We evaluated the robustness of our model predictions of aquatic plant depletion to changes in parameter values. Parameter values were sequentially varied in 10% increments between −100% and +100% of their mean value; a separate simulation was used for each increment. We recorded the range of values over which the model prediction was within ±5% of the observed field data. This conservative value of ±5% was necessary due to the relatively low predicted difference (9%) between the predicted aquatic plant biomasses at the end of the study period for simulations with (169 g DM m^−2^) and without (185 g DM m^−2^) swan grazing. Thus a value of ± ≥10% would not have allowed us to detect differences between scenarios with and without swan depletion of aquatic plants.

### Predicting the effects of a SFA on aquatic plant depletion

To test the effect of the provision of a SFA on the depletion of aquatic plant biomass by swans, we added an additional patch (17000 m^2^) of terrestrial vegetation. We considered the effects of varying three properties of the SFA vegetation, (i) metabolisable energy content, (ii) biomass, and (iii) swan functional response, on the effectiveness of the SFA in preventing grazing of the aquatic plants. We varied metabolisable energy content between 1–15 kJ g^−1^ DM, in 1 kJ g^−1^ DM increments. Metabolisable energy content values were derived as the product of gross energy content and proportional metabolisability. We varied SFA plant biomass values between 200–550 g DM m^−2^, in 25 g DM m^−2^ increments. Our values for SFA metabolisable energy content and biomass represent the full range of values encountered by foraging swan [47], [57], [59]. SFAs may use a range of different plant species [29], each potentially with a different functional response. The functional response describes the relationship between forager intake rate and food biomass. Only two functional responses for mute swans have been reported; values of intake rate for aquatic plants were approximately three-fold higher than for pasture grass [47]. Thus swan intake rate may vary considerably depending on which plant species are present within the SFA. Therefore, to examine how the effectiveness of SFAs varied with the functional response, we sequentially tested 3 values for the intake rate for swans feeding on plants in the SFA. We ran simulations with the pasture grass functional response given previously [47] multiplied by 1.0, 2.0 and 3.0. A separate simulation was run for each combination of metabolisable energy content, biomass and functional response values, and thus 775 simulations were run in total.

## Results

### Testing the model against field data

Our model predictions were typically in close agreement with observed field data (**Table 2**). Initial exploration of the model indicated that results were consistent between simulations due to the deterministic nature of the model. As the swans emigrated before the end of the simulation period, the model predicted a carrying capacity for the study area of 214 swan days, close to the 215 observed in the field. The predicted mean aquatic plant biomass at the end of the 22 day period was 169 g DM m^−2^, which closely matched the observed value of 171 g DM m^−2^. For the period in which the swans were present within the study area (i.e. either present on the in-model river patch or pasture field patch) the mean percentage of time spent by swans on river patches was predicted to be 100%, slightly higher than the 98% observed. Additionally, predicted daily time spent foraging (34%) was within the limits of a time budget study in May in the River Frome (mean ±95% CI = 32±12%; [58]). The percentage of swans which were predicted to starve during the 22 day study period was 0% (i.e. no mortality), which matched field observations [43].

**Table 2:** Five tests of the accuracy of our model predictions, comparing values predicted by our model with observed field data.

Test of model	Predicted value	Observed value	Accuracy
Aquatic plant biomass (g DM m^−2^)	169	171	98.8%
River carrying capacity (swan days)	214	215	99.5%
Swan mortality (%)	0	0	100.0%
Time swans spent feeding (%)	34	32	106.3%
Time swans spent on river (%)	100	98	102.0%

### Model robustness

**Figure 2:**
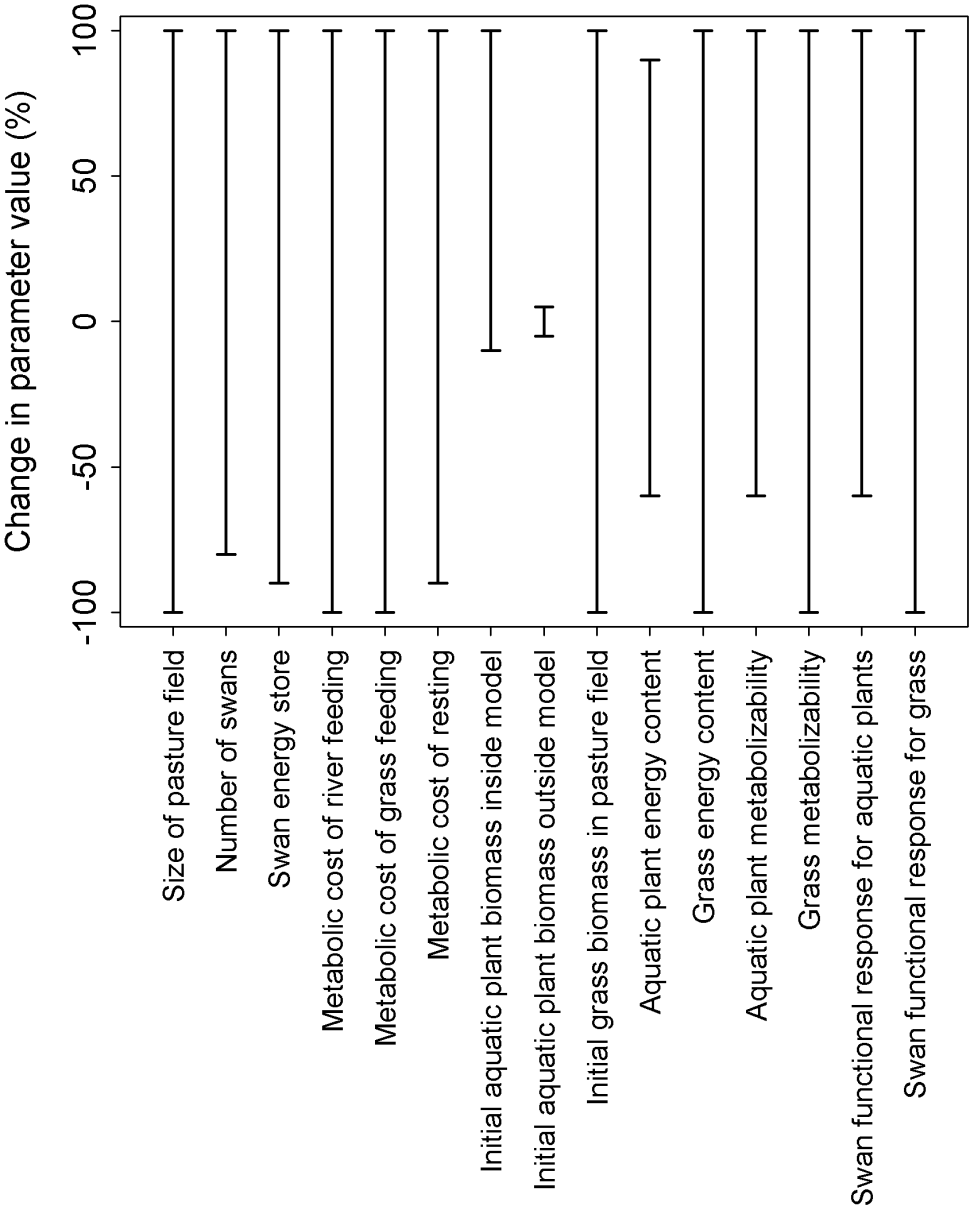
The range of change in parameter values over which the model prediction of aquatic plant biomass was within ±5% of the observed field data.

**Figure 3:**
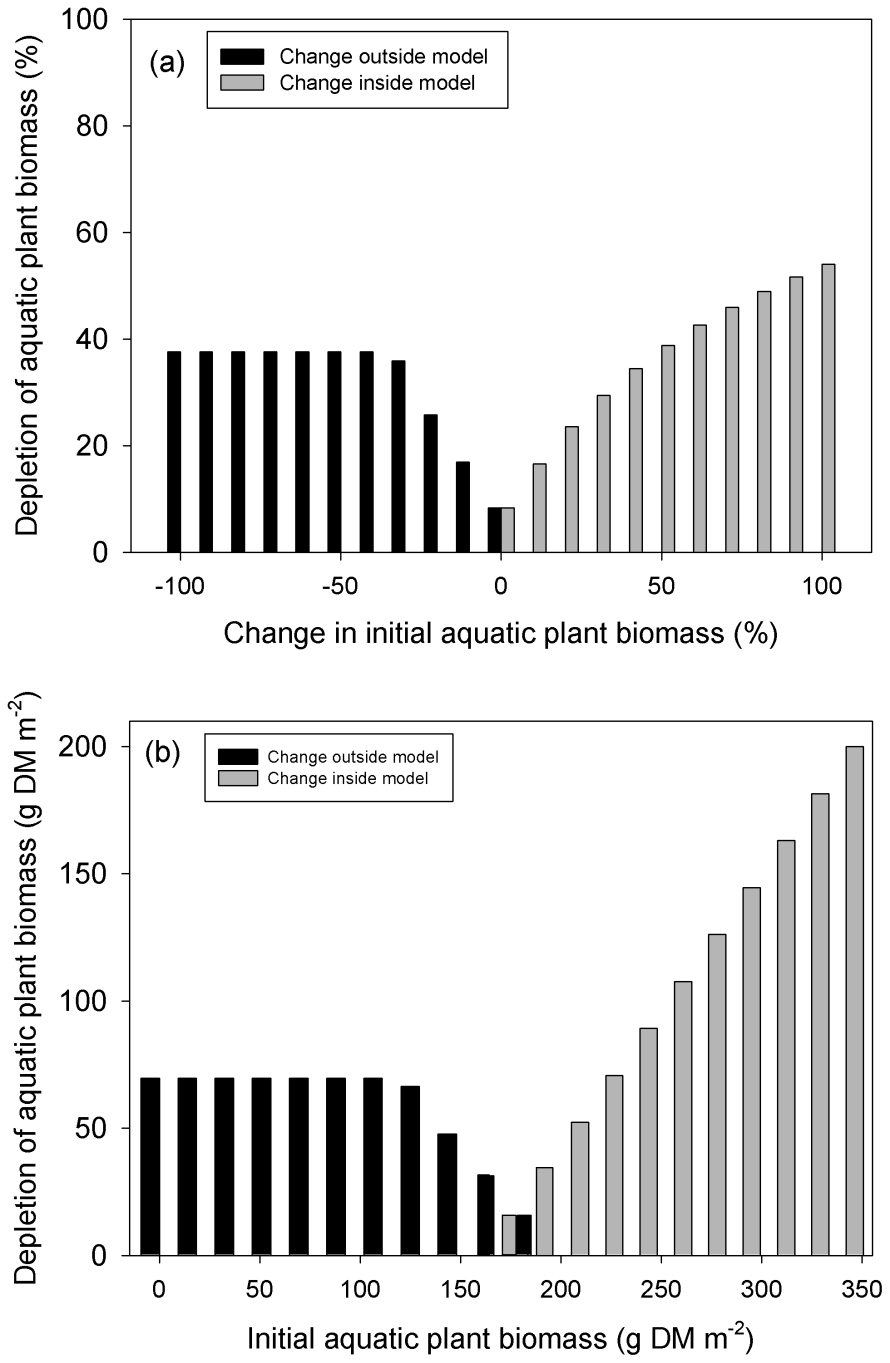
The predicted depletion of aquatic plant biomass in the model river patch after 22 days (i.e. biomass after grazing) varied with the initial aquatic plant biomasses (i) inside the model river patch and (ii) in the river outside of the model. These were based on one-at-a-time changes in aquatic plant biomass, rather than simultaneous changes in both in-model and out-model biomass. Depletion is expressed as (a) percentage, and (b) absolute aquatic plant biomass.

Our model predictions of aquatic plant biomass were robust to large changes (±60%) in the values of 13 out of 15 parameters (**Figure 2**). However, our model predictions were highly sensitive to changes in the initial aquatic plant biomass both within the river patch and outside of the model, as these values strongly determined when the swans should stop grazing within the model and emigrate. Swans would emigrate from the model river patch to the river outside the model during the time step where the aquatic plant biomass of the model river patch decreased below the aquatic plant biomass of the river outside the model. Reducing the initial aquatic plant biomass of the model river patch reduced depletion to 0 g DM m^−2^ as swans emigrated on the first time step and thus did not feed inside the model (**Figure 3**). In contrast, increasing the initial biomass within the model river patch increased depletion, as emigration was delayed due to the greater biomass. Depletion reached 200 g DM m^−2^ for a 100% increase in aquatic plant biomass inside the model river patch. Reduced biomass in the river outside the model increased depletion within the model, up to a maximum of 70 g DM m^−2^ for ≥−40% change.

### Predicting the effects of a SFA on aquatic plant depletion

**Figure 4:**
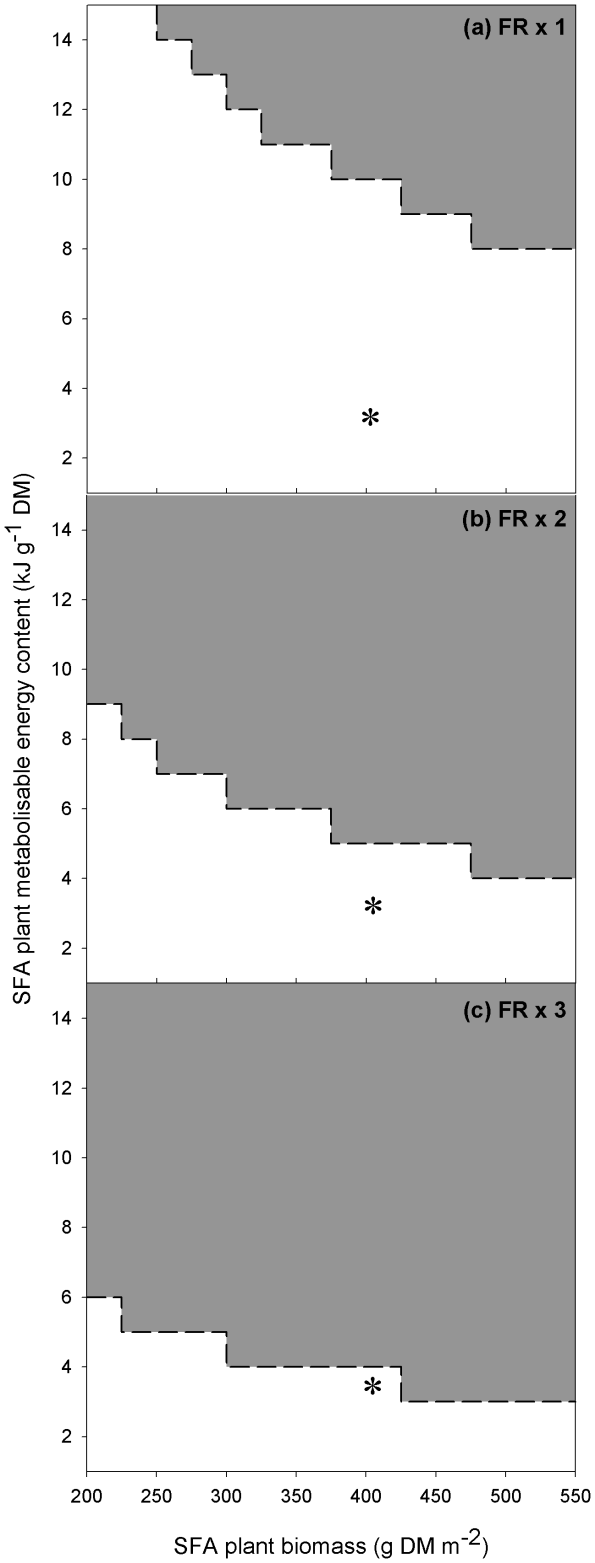
The influence of plant biomass and metabolisable energy content in the sacrificial feeding area (SFA) on aquatic plant biomass in the adjacent river. The dark grey region above the dashed line represents conditions under which aquatic plant biomass was not depleted and thus the SFA was effective. The functional response (FR; food intake rate, g DM hr^−1^) for swans feeding on plants in the SFA was set at (a) ×1.0, (b) ×2.0 and (c) × 3.0 of that previously reported for pasture grass. The symbol * indicates the mean energy and biomass values for SFA pasture grass.

**Figure 5:**
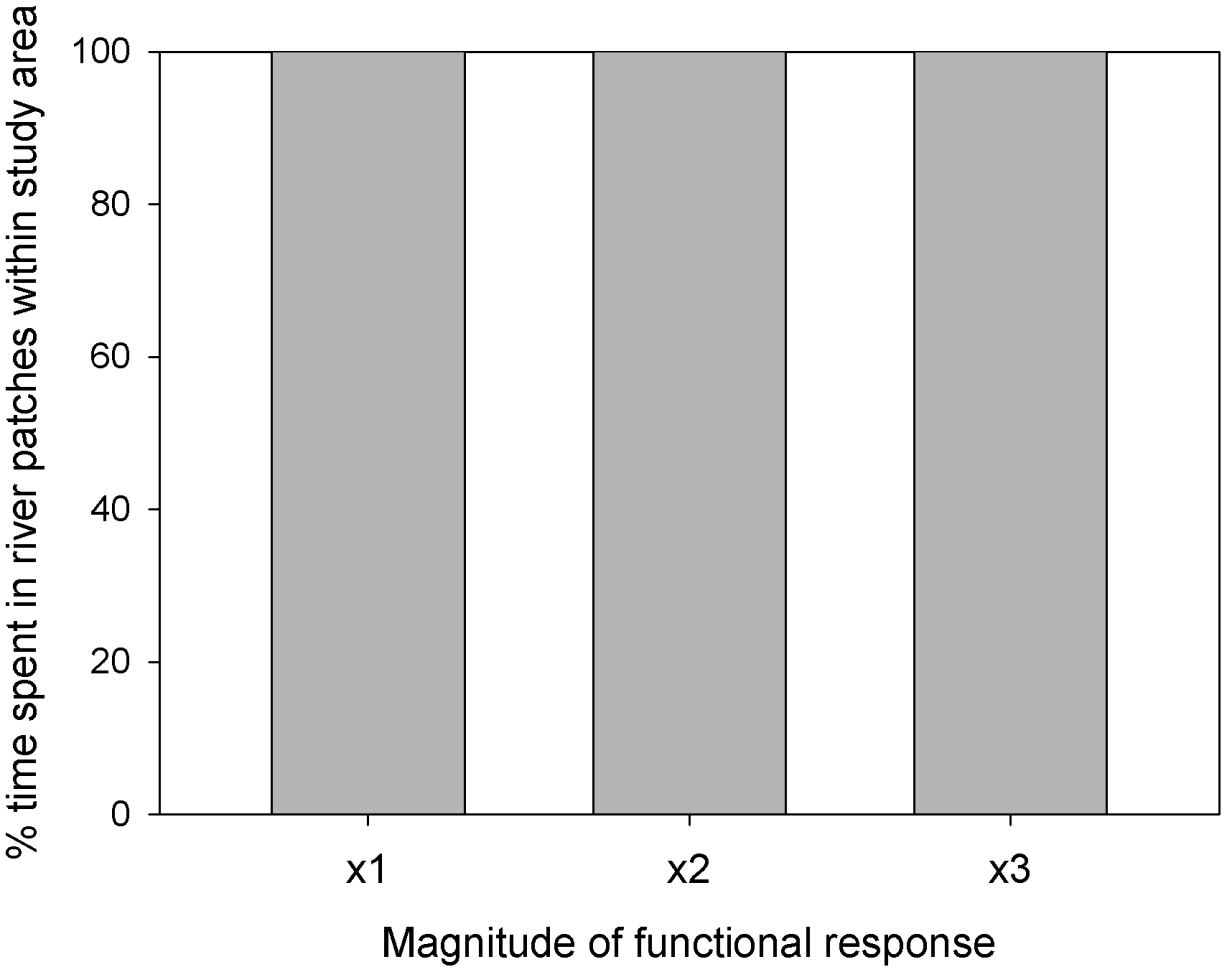
Time spent by the swan population within the river patch, as a percentage of the total time spent within the model study area, for sequential simulations in which the intake rate for SFA vegetation was set to one, two, or three-times the pasture grass functional response, respectively.

For each level of functional response, given a threshold SFA plant biomass and energy content, our model predicted that SFAs could prevent the depletion of aquatic plant biomass (**Figure 4**). Where the SFA was effective at preventing grazing of aquatic plants, aquatic plant biomass was predicted to be 185 g DM m^−2^. Where the SFA was ineffective, aquatic plant biomass was depleted to 169 g DM m^−2^ before the swans emigrated from the model area. Increasing the functional response for the SFA vegetation resulted in lower biomass and energy values required to prevent the depletion of aquatic plant biomass. Where the intake rate for SFA vegetation was set to equal the pasture grass functional response, our model predicted that the SFA would only prevent the aquatic plant depletion at relatively high SFA plant biomass and energy content (**Figure 4a**). To be effective the SFA energy content could be as low as 9 kJ g^−1^ DM given a biomass of 550 g DM m^−2^. Alternatively, an energy content of 15 kJ g^−1^ DM and biomass of 300 g DM m^−2^ was also predicted to be effective. Where the intake rate for SFA vegetation was set to two-times the pasture grass functional response, our model predicted that the SFA would prevent the aquatic plant depletion at lower SFA plant biomass and energy content (**Figure 4b**). To be effective the SFA energy content could be as low as 5 kJ g^−1^ DM given a biomass of 475 g DM m^−2^. Alternatively, an energy content of 10 kJ g^−1^ DM and biomass of 225 g DM m^−2^ was also predicted to be effective. Where the intake rate for SFA vegetation was set to three-times the pasture grass functional response, our model predicted that the SFA would prevent the aquatic plant depletion at lower SFA plant biomass and energy content (**Figure 4c**). To be effective the SFA vegetation energy content could be as low as 3 kJ g^−1^ DM given a biomass of 550 g DM m^−2^. Alternatively, an energy content of 7 kJ g^−1^ DM and biomass of 200 g DM m^−2^ was also predicted to be effective. However, given the known values for grass metabolisable energy (3.3 kJ g^−1^ DM) and biomass (406.0 g DM m^−2^), swans were predicted to always use river habitat whilst in the study area, even when the intake rate for SFA vegetation was set to three-times the pasture grass functional response (**Figure 5**).

## Discussion

In this study, we demonstrated how behavioural ecology can be used to inform conservation and wildlife management, by evaluating how the provision of a sacrificial feeding area of vegetation could divert a population of mute swans away from an adjacent river and thus prevent grazing damage to aquatic plants. Whilst previous studies have examined the effects of SFA provision on grazing conflicts [27], [28], our use of an individual-based model allows us to explicitly link SFA vegetation properties to forager energetics and decision-making processes [34]. An examination of how changes in the factors which regulate SFA profitability to foragers, such as plant biomass, nutritional quality and forager functional response, offers a detailed, predictive understanding of the circumstances under which SFAs will be effective in attracting foragers and thus prevent grazing conflicts [60], [61], [62]. Our model predicted that SFA vegetation was required to exceed threshold values for food quantity and nutritional quality, and allow a sufficiently high intake rate, to attract foraging swans away from the river. Thus only limited support was found for our hypothesis that the provision of terrestrial SFA vegetation could prevent the depletion of aquatic plan biomass in an adjacent river. The threshold for each factor was dependent on the value of the other two factors, which indicates the need to consider the range of properties which determine the net rate of energy gain available to the forager. Combined increases in SFA vegetation biomass and nutritional quality facilitated a switch from river to SFA at lower values of biomass and nutritional quality than increases in either factor in isolation. These thresholds were set by the net energy gain available to swans feeding on river vegetation; a wide range of animal species have been shown to select foraging habitat so as to maximise their net rate of energy gain [18], [19], [20]. Thus the principles that underpin our model predictions are likely to hold true across a range of different resource-consumer interactions, emphasising the generality of our approach to the evaluation of strategies for resolving wildlife management problems.

Given the known values of biomass, energy and swan intake rate for pasture grass during summer, our model predicted that an SFA of pasture grass would be insufficient to prevent depletion of aquatic plants. In order to realise the potential of SFAs for managing herbivores, we required data on herbivore foraging ecology, such as the functional responses to different plants, and plant properties such as biomass dynamics and nutritional quality. Our study highlights the value of collecting such basic ecological data. We currently lack the required data on the characteristics of alternative terrestrial plant species to pasture grass which could be used in an SFA, such as oilseed rape (*Brassica napus* L.), wheat (*Triticum spp*.) and clover (*Trifolium spp*.). Such crops would have to be sown during the early summer period so that their early-growth stage, which are most attractive to waterfowl, coincides with the period when SFA vegetation is required. Waterfowl metabolisability is known to be greater for oilseed rape than grass [63], however, waterfowl intake rates for these crop types have not been quantified. Despite the lack of available data for waterfowl, studies of the relative intake rates, metabolisability and energy content for mammalian herbivores feeding on different crop types can give some indication of their suitability for SFAs. For example, sheep feeding on clover obtained a maximum intake rate that was 1.7 times greater relative to pasture grass [64], but metabolisability did not differ despite a 1.5 times greater energy content for clover [65]. Assuming that swans feeding on clover with a 1.5 times greater energy content could achieve a similar 1.7 times greater intake rate compared with pasture grass feeding, swan energy gain during the May-June period would be 119% greater than would be gained by feeding on aquatic plants. Clearly, further work to quantify swan feeding parameters on these alternative crop types is needed to assess their utility as SFA crops with more confidence. A strength in our approach is that we have identified the characteristics, in terms of biomass, energy content and herbivore intake rate, which SFA plant species must have in order to successfully alleviate the grazing conflict. Thus a lack of data on alternative food resources need not prevent the evaluation of the criteria required for successful management.

It is important to evaluate the potential limitations of any management strategy, in particular for sacrificial feeding areas, which have met with mixed success in field trials [23], [24], [26]. The creation of SFAs will increase food availability within the landscape and thus where food availability limits survival the provision of additional food could increase individual survival and productivity, and thus population size [66], [67], [68]. The duration and timing of SFA food availability are critical factors, as the additional food of an SFA will affect survival and productivity only if supplied for sufficient time during the period of low natural food availability which for most temperate species is winter [34], [69]. Where other factors limit numbers of a species, such as predation, habitat availability or disease, the addition of supplementary food is unlikely to result in increased numbers. Indeed there have been numerous studies which have found that the experimental provision of additional food resources did not result in increased numbers [70], [71]. The super-abundance of vegetation within many modern temperate landscapes, where agriculture is the dominant land use, means that for many vertebrate herbivore species food does not currently limit survival [69]. Therefore, the short-term provision of additional food is unlikely to increase survival or productivity of generalist herbivore species such as mute swans. Previous research has indicated that the number of territories, not food abundance, currently limits swan breeding population size in mute swan populations within our study area [13]. Furthermore, there is no evidence of increased population size in response to the provision of SFAs for mute swans in agricultural land in Scotland [48]. However, SFA provision could conceivably result in a small localised increase in swan numbers if non-breeding vagrants, which are known to move in and out of our study area [13], are more likely to remain within the study area due to greater food abundance resulting from SFA provision. Ultimately, the purpose of SFAs is to relocate undesirable consumption to an area where it can be tolerated, rather than to prevent consumption within the landscape. SFAs are unlikely to be suitable for species whose presence anywhere in the landscape is undesirable, such as invasive species. The availability of suitable land for SFAs, connectivity with the site of conflict, and the dispersal ecology of the target species, must all be carefully considered. SFAs are likely to be most effective for animals which can disperse efficiently between feeding areas, and thus appear well suited to resolving conflicts with waterfowl [22].

Using behavioural ecology to understand the requirements of successful wildlife management can allow such management to become predictive, rather than reactionary, which has been a longstanding aim of wildlife managers [72]. By considering changes in the distribution of food resources within the landscape, further research should aim to forecast spatiotemporal patterns in consumer-resource interactions at the landscape scale and thus predict where wildlife impacts and conservation conflicts could occur. Our modelling approach shows how such predictions can be made and evaluated.
